# Pharmacokinetics of Hydroxytyrosol and Its Sulfate and Glucuronide Metabolites after the Oral Administration of Table Olives to Sprague-Dawley Rats

**DOI:** 10.1021/acs.jafc.3c06431

**Published:** 2024-01-17

**Authors:** Ivana Kundisová, Helena Colom, M. Emília Juan, Joana M. Planas

**Affiliations:** † Grup de Fisiologia i Nutrició Experimental, Departament de Bioquímica i Fisiologia, Facultat de Farmàcia i Ciències de l’Alimentació and Institut de Recerca en Nutrició i Seguretat Alimentària (INSA-UB, María de Maeztu Unit of Excellence), Universitat de Barcelona (UB) and Food Innovation Network (XIA), Av. Joan XXIII 27-31, 08028 Barcelona, Spain; ‡ Grup de Farmacocinètica, Famacodinàmia i Farmacogenòmica Poblacional, Departament de Farmàcia i Tecnologia Farmacèutica, i Fisicoquímica, Facultat de Farmàcia i Ciències de l’Alimentació, Universitat de Barcelona (UB), Av. Joan XXIII 27-31, 08028 Barcelona, Spain

**Keywords:** hydroxytyrosol, hydroxytyrosol glucuronide, hydroxytyrosol sulfate, noncompartmental analysis, Arbequina table olives

## Abstract

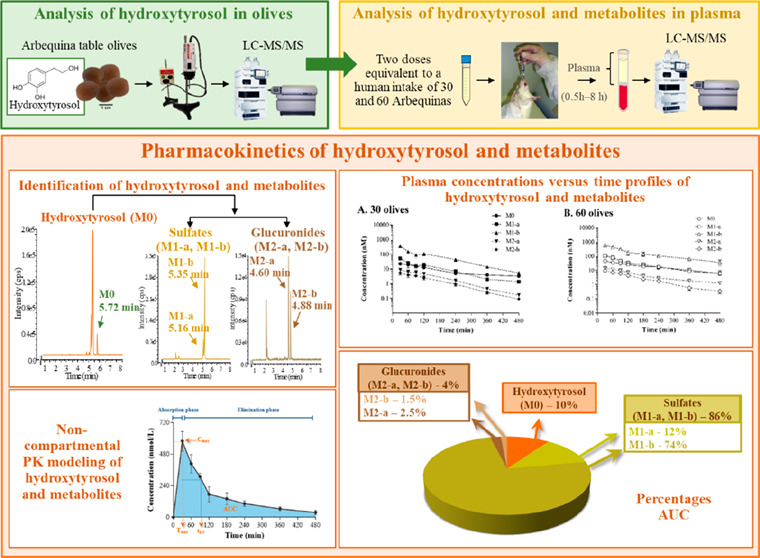

The pharmacokinetics (PK) of hydroxytyrosol and its metabolites were characterized following oral administration to Sprague-Dawley rats of 3.85 and 7.70 g of destoned Arbequina table olives/kg. Plasma samples were analyzed using a fully validated method consisting of liquid extraction followed by liquid chromatography-tandem mass spectrometry (LC-MS/MS). Noncompartmental PK analysis of hydroxytyrosol demonstrated linear PK between doses of 2.95 and 5.85 mg hydroxytyrosol/kg. Half-life was approximately 2.5 h, while mean residence time was around 4 h. Clearance occurred by conversion to two sulfate and two glucuronide conjugates. The area under the plasma concentration–time curve (AUC) ratios of metabolites versus parent hydroxytyrosol was approximately 7–9-fold for the sulfate and below 0.25 for the glucuronide, indicating sulfation as the predominant metabolic pathway. Despite extensive metabolism, hydroxytyrosol remained in plasma for up to 8 h with AUCs of 4293 and 8919 min·nmol/L for the doses of 3.85 and 7.70 g/kg, respectively. Therefore, table olives provide a more sustained plasma profile than other foods containing hydroxytyrosol, which may enhance its health-protecting activities.

## 1 Introduction

Numerous investigations have established that bioactive compounds from certain foods exert health-protecting properties and have been associated with a lower prevalence of degenerative diseases.^1^ This is the case of extra virgin olive oil (EVOO), whose content of hydroxytyrosol has attracted significant scientific interest owing to its wide range of beneficial effects on health.^2−6^ In this sense, the European Food Safety Authority (EFSA) specified in the EU Regulation no. 432/2012, that a daily intake of 20 g of EVOO containing at least 5 mg of hydroxytyrosol and derivatives contributes to the protection of blood lipids from oxidative stress and consequently recognizes the capacity of this phenolic compound to reduce cardiovascular risk at a regular dose.^7^ Thus, ensuring the amount of hydroxytyrosol provided by dietary sources is important. A recent study has established that although the average hydroxytyrosol content in commercially available European EVOO is 5.2 μg/g,^8^ the dietary intake for the adult population in Europe was only 1.97 ± 2.62 mg/day.^8^ These values are below the EFSA recommendations,^7^ and to achieve them, it would be necessary either to enrich EVOO with hydroxytyrosol, take supplements, or complement the diet with a regular intake of table olives. Bearing in mind that the reported average content of hydroxytyrosol in different varieties ranged from 384 to 764 mg/kg,^9−11^ the inclusion of this food on a regular basis could reinforce the amount of this polyphenol provided by EVOO.

Although table olives emerge as an alternative dietary source of hydroxytyrosol to olive oil, no *in vivo* studies have been carried out that provide a quantitative evaluation of the pharmacokinetics (PK) from that specific matrix. Up to now, different investigations have assessed the plasma concentrations of hydroxytyrosol in humans after the oral intake of EVOO,^12−14^ olive leaf extract,^15,16^ and pure standard^17^ or in rats after the oral administration of the pure compound^18−22^ and from olive cake.^23^ However, the predictability of hydroxytyrosol bioavailability from table olives based on these studies remains limited given the influence of food matrix on release and accessibility of phenolic compounds.^24^ Concerning table olives, there are two studies in the literature describing the plasma profile after their intake in healthy volunteers^25,26^ while in rats there is only one report determining the plasma concentrations at 0 and 30 min postadministration.^11^ Therefore, our study evaluated the pharmacokinetics of hydroxytyrosol in Arbequina table olives in rats and characterized the plasma hydroxytyrosol phase II metabolites. Although absorption and metabolism of hydroxytyrosol and derivatives from olive oil have previously been reported, the novelty of this work consists of thoroughly characterizing the complete pharmacokinetic profile of hydroxytyrosol and conjugates from table olives. To achieve this aim, two doses of 3.85 or 7.70 g of destoned olives/kg, containing 764 ± 9.47 mg of hydroxytyrosol/kg of olives, were administered by gavage. Then, male Sprague-Dawley rats received a dose of hydroxytyrosol of 2.95 or 5.89 mg/kg. The doses administered to the rats are equivalent to the human consumption of 30 and 60 Arbequina table olives and were selected because the intake of 30 olives was double to one serving according to the Mediterranean diet pyramid.^27^ Moreover, this dosage is relevant to health because the administration of 3.85 g/kg to spontaneously hypertensive rats decreased blood pressure,^28^ being the antihypertensive effect associated with changes in the concentration of angiotensin II, malondialdehyde as well as gut microbiota remodeling.^29^ Our results have demonstrated that hydroxytyrosol provided in table olives, although extensively metabolized, remained in plasma during 8 h. Therefore, table olives can be considered an efficient vehicle to deliver hydroxytyrosol, thus, emerging as a functional food.

## 2 Materials and Methods

### 2.1 Chemicals and Reagents

Hydroxytyrosol was purchased from Seprox BIOTECH (Madrid, Spain). 2-(3-Hydroxyphenyl) ethanol (internal standard, I.S.) and l-ascorbic acid were obtained from Sigma-Aldrich (Tres Cantos, Spain). Acetone, acetonitrile, 2-propanol, methanol, and tetrahydrofuran were obtained from Panreac Química SLU (Castellar del Vallés, Spain). Ethyl acetate was supplied from J.T. Baker (Deventer, Netherlands), and glacial acetic acid was from Merck (Darmstadt, Germany). The chemicals used were analytical grade, and the solvents were LC-MS grade. Ultrapure water obtained using a Milli-Q water purification system (18 mΩ) (Millipore, Milan, Italy) was employed in all experiments.

### 2.2 Animals

Male adult Sprague–Dawley rats with a body weight of 300–350 g were from the Animal House Facility at the Facultat de Farmàcia i Ciències de l’Alimentació of the Universitat de Barcelona. Animals were kept in groups of two rats per cage, under controlled conditions of a light–dark cycle of 12 h, with a relative humidity of 50 ± 10% and temperature maintained at 22 ± 2 °C. Animals were given a standard solid diet (2014 Teklad Global 14%, Envigo Rms Spain SLU, Sant Feliu de Codines, Spain) and water *ad libitum*. All experiments were performed in the morning to minimize the influence of circadian rhythms in animals deprived of food overnight but with free access to water. Prior to the experiments, the solid diet, as well as blank plasma, were analyzed, and no traces of hydroxytyrosol were found. Terminal anesthesia was performed by intraperitoneal injection of 90 mg/kg of ketamine (Imalgene 1000, Merial Laboratorios SA, Barcelona, Spain) and 10 mg/kg of xylazine (Rompun 2%, Química Farmacéutica Bayer SA, Barcelona, Spain).

All animal manipulations were performed in full compliance with the guidelines established by the European Community for the care and management of laboratory animals and were approved by the Ethics Committee of Animal Experimentation of the Universitat de Barcelona (CEEA-UB ref 373/12) and the Generalitat de Catalunya (ref 9468).

### 2.3 Arbequina Table Olives

Table olives from the Arbequina variety (Cooperativa del Camp, Maials, Lleida) were cultivated in Ribera d’Ebre (Tarragona) in orchards with drip irrigation. The fruits of *Olea europaea* L. were harvested in the green-yellow stage of maturation and in perfect sanitary conditions during the season 2015/2016. The olives were debittered by natural fermentation in an 8% (w/v) NaCl brine solution for over 2 months. Subsequently, the olives were rinsed and transferred to a final brine composed of 3.5% (w/v) NaCl.

### 2.4 Dose Selection and Preparation

For the selection of the dose, a human intake of 30 Arbequina table olives was considered because it corresponded to a daily consumption of two portions as recommended by the Mediterranean diet pyramid.^27^ The translation of the dose from humans to animals was performed using the body surface area normalization method described by Reagan-Shaw et al.^30^ Therefore, the calculated dose to be administered to the rats was 3.85 g of destoned olives/kg, and the group was named “30 olives” because it was equivalent to a consumption of 30 Arbequina table olives by a 60 kg person. A double dose of 7.70 g/kg was administered to the “60 olives” group.

The doses of Arbequina table olives were prepared as a homogeneous suspension. Hence, the stones were removed from the olives, and the edible part was mixed with Milli-Q water and then carefully homogenized with a Polytron blender (Kinematica AG, Lucerne, Switzerland) equipped with a 20 TS arm. The homogenization procedure involved six 30 s long blending cycles carried out at a speed set at 5, with 1 min pauses in between cycles to avoid excess heating.

### 2.5 Experimental Design

The experiments were performed using Sprague–Dawley rats (*n* = 13) randomly divided into two groups, the 30 olives group (*n* = 6) that received the dose of 3.85 g/kg and the 60 olives group (*n* = 7), which was given the dose of 7.70 g/kg. The freshly prepared homogeneous suspensions were given to overnight fasted rats by oral gavage (18-gauge × 76 mm, ref FFSS-186-76, Instech Laboratories, Inc., Plymouth Meeting, PA). The volume of administration was 10 mL/kg. After the single administration of Arbequina table olives, blood was obtained at 30, 60, 90, 120, 240, 360, and 480 min from each animal. Samples were withdrawn from the lateral saphenous vein and placed into Microvette CB 300 K_2_ EDTA-K_2_ (Sarstedt, Granollers, Spain) coated tubes.^31^ At each time point, 0.40 mL of blood was collected, meaning that a maximum of 2.4 mL was obtained from each animal. This volume represents 10% of all circulating blood and does not affect the hematocrit.^32^ Blood corresponding to 480 min was taken by a cardiac puncture with the animal under terminal anesthesia. Plasma was immediately obtained by centrifugation at 1500*g* at 4 °C for 15 min (Centrifuge Megafuge 1.0R, Heraeus, Boadilla, Spain) and kept at −20 °C until analysis.

### 2.6 Determination of Hydroxytyrosol and Its Metabolites from Rat Plasma by LC-MS/MS

Hydroxytyrosol and its metabolites in plasma were determined by liquid–liquid extraction followed by LC-MS/MS analysis as previously described.^11^ Briefly, 200 μL of plasma was mixed with 10 μL of freshly prepared 10% ascorbic acid followed by 10 μL of 0.5% acetic acid and 10 μL of 2-(3-hydroxyphenyl) ethanol (10 μM, I.S.). Then, 2 mL of ethyl acetate was added to the samples that were vigorously mixed in a vortex (5 min), placed into an ultrasonic bath (10 min), and centrifuged at 1500*g* at 4 °C (15 min) (Centrifuge Megafuge 1.0R). A second extraction of the pellet with 2 mL of ethyl acetate was carried out. Subsequently, the two pooled supernatants were evaporated to dryness, reconstituted with 100 μL of 80% methanol, placed into amber vials, and immediately analyzed by LC-MS/MS.

An Agilent 1260 liquid chromatograph (Agilent Technologies, Santa Clara, USA) coupled to a QTRAP 4000 mass spectrometer (AB Sciex, Toronto, Canada) equipped with a Turbo V electrospray ionization (ESI) source was used for the determination of hydroxytyrosol and its metabolites. Analyst software, version 1.6.2. (AB Sciex) operated the instrument and was employed for data analysis. The equipment was located at the Scientific and Technological Centre of the Universitat de Barcelona (CCiTUB).

Injections of 2 μL of each sample were performed by an automated autosampler that maintained vials at 10 °C to avoid degradation. Chromatographic separation of hydroxytyrosol and its metabolites was performed in a Zorbax Eclipse XDB-C18 reversed-phase column (150 mm × 4.6 mm, 5 μm) protected with a guard cartridge of the same material (Zorbax Eclipse XDB-C18, 12.5 mm × 4.6 mm, 5 μm) with the temperature set at 30 °C. The mobile phase consisted of phase A formed by Milli-Q water with 0.025% acetic acid and phase B containing acetonitrile with 5% acetone delivered at a flow of 0.8 mL/min. The following gradient elution was used: 0 min, 95% A and 5% B; 1 min, 90% A and 10% B; 10 min, 35% A and 65% B; 10.5 min, 0% A and 100% B. Solvent B was maintained at 100% for 5 min to prevent carryover prior to returning to initial conditions. A 6 min delay was programmed before the next injection to ensure the equilibration of the system. Moreover, the injector needle was washed with 1:1:1 (v/v) 2-propanol, tetrahydrofuran, and Milli-Q water to avoid further carryover.

**Figure 1:**
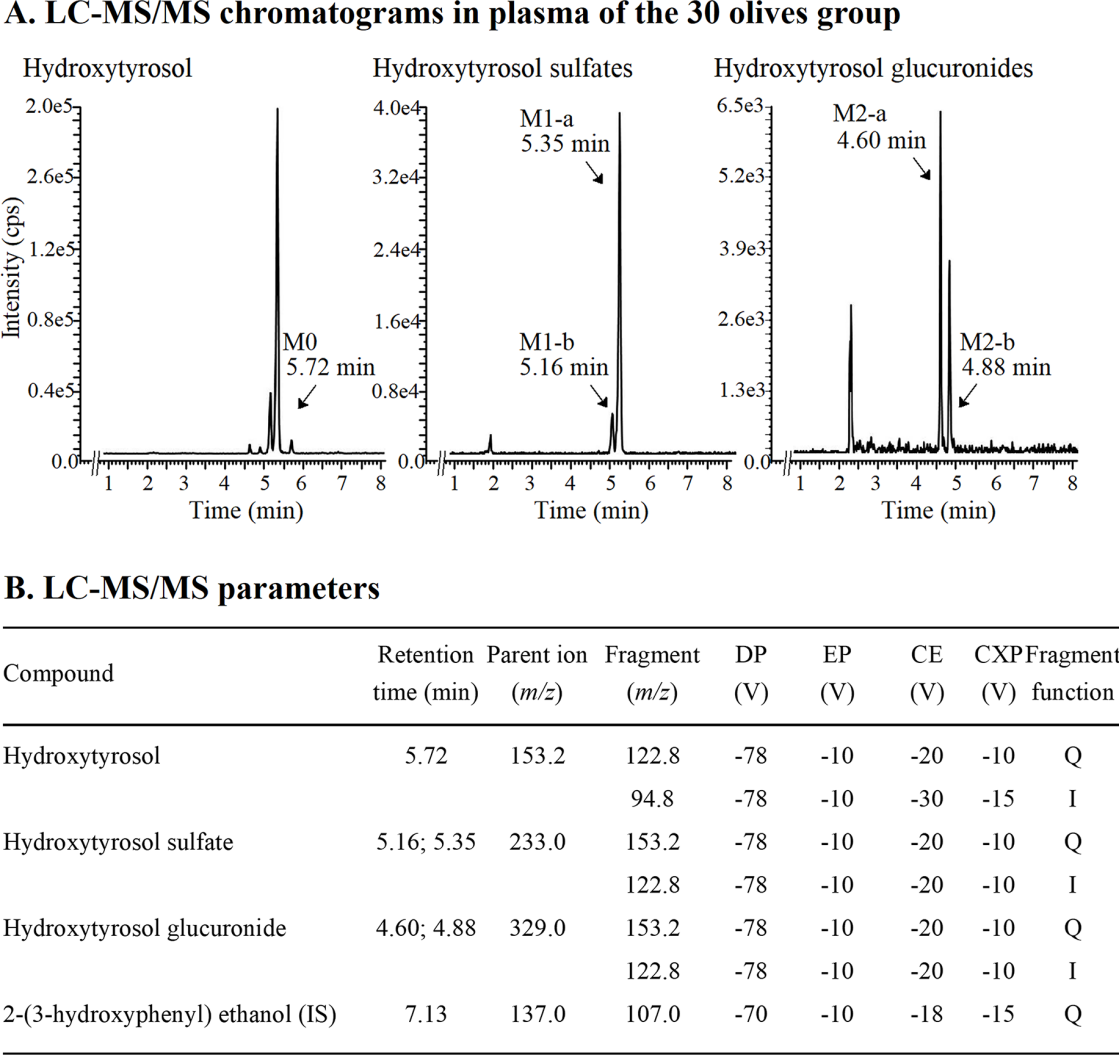
(A) Representative liquid chromatography-tandem mass spectrometry (LC-MS/MS) chromatograms of hydroxytyrosol and its metabolites in plasma obtained 30 min after the oral administration to Sprague-Dawley rats of Arbequina table olives containing hydroxytyrosol at 2.95 mg/kg (30 olives group). (B) Multiple reaction monitoring (MRM) parameters corresponding to hydroxytyrosol and its metabolites set or obtained by LC-MS/MS. The quantification (Q) and qualification (I) transitions were monitored for the hydroxytyrosol and its metabolites while only one transition was employed for the internal standard (I.S.). DP, declustering potential; EP, entrance potential; CE, collision energy; CXP, collision cell exit potential.

The ESI source, operating in negative mode, was set as follows: temperature, 600 °C; curtain gas (N_2_), 25 arbitrary units (au); ion source gas 1 (source heating gas, N_2_); 50 au; ion source gas 2 (drying gas, N_2_); 50 au, and ionization spray voltage, −3500 V. The MS analysis was performed in multiple reaction monitoring (MRM) mode and the specific parameters are shown in Figure 1. Within each analytical run, a full set of calibration standards was injected including a reagent blank and blank plasma.

The plasmatic concentrations were calculated by the interpolation of the peak area ratio of hydroxytyrosol versus I.S. on a calibration curve. Calibration standards were constructed with blank plasma obtained by cardiac puncture from overnight fasted rats that had never received either table olives or hydroxytyrosol. Then, 190 μL of blank plasma was spiked with 10 μL of working standards at 0, 200, 500, 1000, 2000, 3000, and 5000 nmol/L to obtain the final concentrations of 0, 10, 25, 50, 100, 150, and 250 nmol/L. Metabolites were identified with the *m*/*z* indicated in Figure 1 and were assumed to possess a LC-MS/MS response similar to that of hydroxytyrosol. Hence, the concentrations of the sulfate and glucuronide derivatives were quantified using the standard curve of the parent compound. Results were expressed in nmol per liter of plasma (nmol/L). The method was validated following the EMA guidelines^33^ at six different concentrations ranging from 0 to 250 nmol/L analyzed on three different days. Validation results indicated that the analytical method is linear (*R*^2^ > 0.998), precise (CV < 15%), with satisfactory recovery (98.4 ± 1.64%), absence of matrix effect (96.7 ± 2.75%), no carry-over, and adequate sensibility with a limit of quantification (LOQ) of 0.2 nmol/L.

### 2.7 Pharmacokinetic Analysis of Hydroxytyrosol and Its Metabolites in Rat Plasma

Individual PK of hydroxytyrosol and its metabolites were estimated from the plasma concentrations versus time profiles of each animal, through a noncompartmental analysis (NCA) with Phoenix-64 (Build 8.3.4.295, Certara, Princeton, N.J., USA). All concentrations of each pharmacokinetic profile were considered for NCA because the values obtained were above the LOQ. Peak plasma concentration (*C*_max_) and time to peak plasma concentration (*T*_max_) were determined by visual inspection of the pharmacokinetic profiles. The apparent elimination rate constant (λ_*z*_) was estimated from the terminal slope of the semilogarithmic concentration–time curve. The apparent elimination half-life (*t*_1/2λ*z*_) was calculated as *t*_1/2z_ = 0.693/λ_*z*_. The area under the plasma concentration–time curve from time zero to the last experimental time (AUC_t_) with analyte concentrations above the LOQ, was calculated by the linear-log trapezoidal rule. The area from time zero to infinity (AUC_0–∞_) was calculated by adding to the AUC_t_ value the extrapolated area calculated as the ratio between the predicted concentration (*C*_t_) at the last sampling time with concentrations above the LOQ and λ_*z*_. The mean residence time (MRT_0–∞_) was given by the ratio of the area under the first-moment curve (AUMC_0–∞_) to the area under the zero-moment curve (AUC_0–∞_). The ratio indicating the relative exposure of each metabolite with respect to hydroxytyrosol (AUC_0–∞_m/AUC_0–∞_) was also calculated for all of the metabolites. The actual clearance (Cl) and volumes of distribution (V) could not be determined because hydroxytyrosol was not administered intravenously and the bioavailability (F) was unknown. Consequently, the apparent clearance was calculated as Cl/F = D/AUC, the apparent steady-state distribution volume (Vss/F) was estimated as Vss/F = MRT·Cl/F, and the apparent distribution volume associated with the terminal phase of the plasma concentration–time curve (Vd_area_/F) was obtained as Vd_area_/F = *D*/AUC·λ_*z*_. Moreover, the fraction of hydroxytyrosol converted to each metabolite (fm) was unknown due to the lack of intravenous data on both the parent compound and the derivatives. Therefore, only apparent parameters could be estimated (CI/F·fm; *V*/F·fm).

### 2.8 Statistical Analysis

Descriptive statistics of individual concentrations of hydroxytyrosol and its metabolites at each experimental time were calculated and presented as the mean ± standard error. Geometric means (95% CIs) of the exposure and PK parameters were calculated from all the animals, except for *T*_max_ which is given as the median with its minimum and maximum value. The log-transformed normalized by dose values of exposure metrics (*C*_max_/Dose and AUC_0–∞_/Dose; Dose expressed as nmol), AUC_0–∞_m/AUC_0–∞_ ratios, and the remaining PK parameters (Cl/F, Vss/F, Vd_area_/F, *t*_1/2λ*z*_, and MRT), were compared between doses to investigate a potential dose-dependent or nonlinear pharmacokinetic behavior. An unpaired-Student *t*-test was applied in all the cases except for *T*_max_ comparisons which were analyzed with a nonparametric Wilcoxon signed-rank test. The log-transformed values of the terminal phase slopes of the plasma-concentration profiles between hydroxytyrosol and its metabolites were also compared by a two-way analysis of variance (ANOVA) considering the dose and the compound as fixed factors. The interaction between both factors was initially considered and then removed if it was not statistically significant. The statistical significance level was set at α = 0.05. All of the statistical analyses were carried out using IBM SPSS Statistics for Windows, version 25.0 (IBM Corp., Armonk, NY, USA).

## 3 Results

### 3.1 Hydroxytyrosol in Tables Olives

Arbequina table olives possessed a weight per fruit of 1.75 ± 0.06 g (*n* = 10) and a destoned olive weight of 1.25 ± 0.04 g. The dry weight of the edible part was 0.41 ± 0.02 g. The content of hydroxytyrosol was analyzed by LC-MS/MS following the method previously described^11^ and was found to be 764 ± 9.47 mg/kg of destoned olives (*n* = 3). The doses of hydroxytyrosol administered corresponded to 2.95 mg/kg (19.1 μmol/kg) and 5.89 mg/kg (38.2 μmol/kg) in the 30 and 60 olive groups, respectively.

### 3.2 Identification of Hydroxytyrosol and Its Metabolites in Rat Plasma

Hydroxytyrosol was identified in rat plasma by its retention time (5.72 min) and by monitoring the fragmentation pattern of the deprotonated molecular ion and the specific fragment recorded in MRM mode (*m*/*z* 153.2/122.8 Da). The analysis of the chromatograms obtained from plasma allowed the identification of hydroxytyrosol, which was found at all sampling times in both doses administered.

Figure 1 shows the representative LC-MS/MS extracted ion chromatograms corresponding to plasma obtained 30 min after the oral administration of Arbequina table olives at doses equivalent to a human intake of 30 and 60 table olives. At both doses, four additional peaks were found in the chromatograms of hydroxytyrosol (Figure 1). In a targeted analysis, the two larger peaks were identified as sulfate derivatives (M1-a and M1-b), while the two smaller peaks corresponded to glucuronide metabolites (M2-a and M2-b). In the case of hydroxytyrosol sulfates that appeared at 5.16 (M1-a) and 5.35 min (M1-a), an increase in molecular weight of 80 Da was found, thus the product ion was detected in negative mode at *m*/*z* 233.0. This metabolite was analyzed by two transitions, the first one at *m*/*z* 233/153 (quantification transition) and the second one at *m*/*z* 153.0/122.8 (qualifier transition). An increase of 176 Da in the mass of the parent compound was observed for glucuronide metabolites; therefore, the product ion was detected in the negative mode at the *m*/*z* 329.0. Glucuronide metabolites, which eluted at 4.60 (M2-a) and 4.88 min (M2-b) were analyzed by two transitions, the first at *m*/*z* 329/153 (quantification transition) and the second one at *m*/*z* 153.0/122.8 (qualifier transition).

Blank plasma samples were checked for the presence of hydroxytyrosol and its metabolites, and no traces of any of the investigated compounds were observed.

### 3.3 Pharmacokinetic Analysis

#### 3.3.1 Hydroxytyrosol

**Figure 2:**
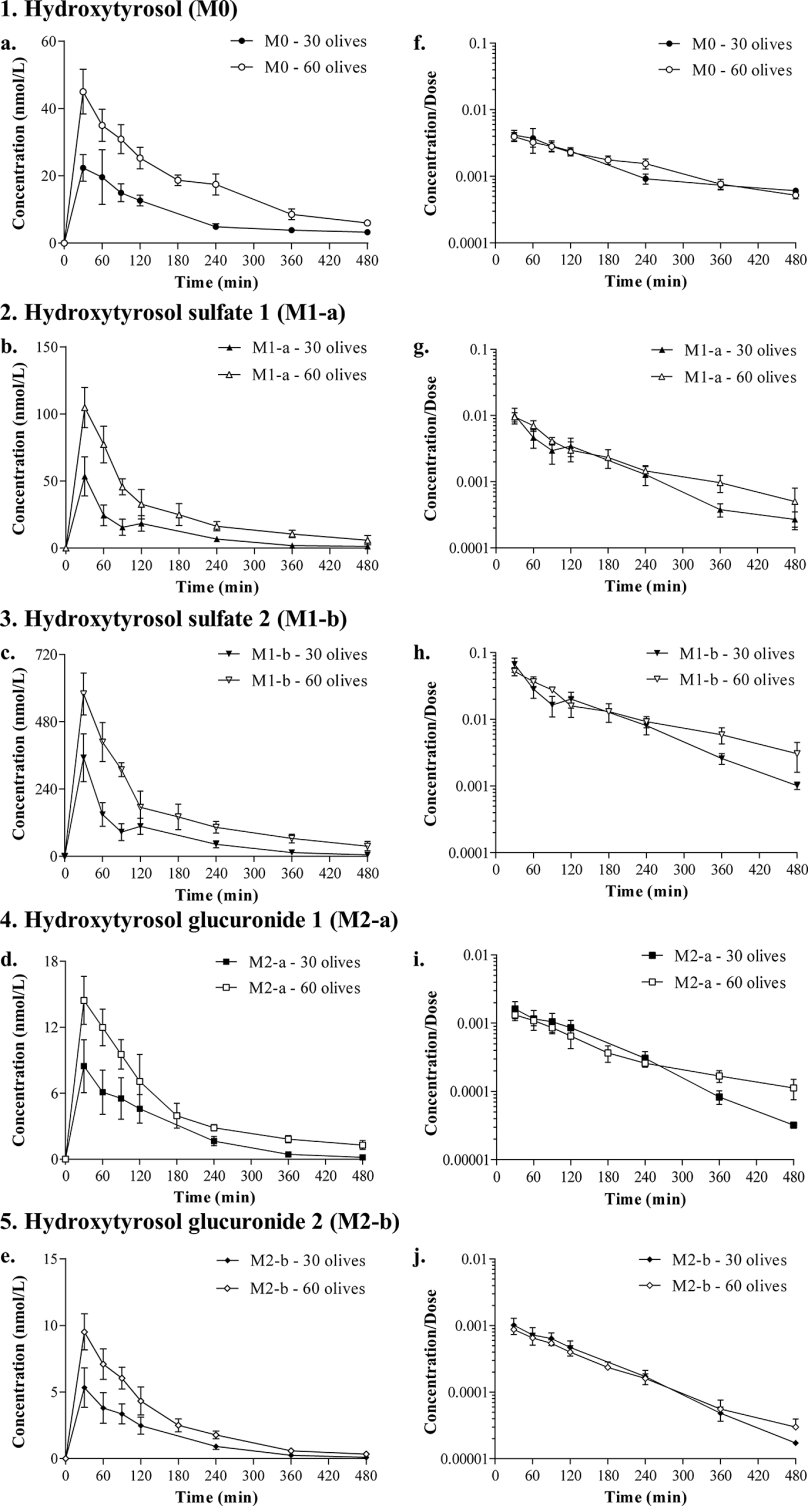
Plasma concentrations versus time profiles of hydroxytyrosol (a) and its metabolites (b–e) obtained after the oral administration to Sprague-Dawley rats of Arbequina table olives containing hydroxytyrosol at 2.95 mg/kg (30 olives group) and 5.89 mg/kg (60 olives group). The ratio of the plasma concentrations of hydroxytyrosol (f) and metabolites (g–j) with the dose of hydroxytyrosol was represented in a semilogarithmic scale to assess the linearity between the two doses. Results are expressed as means ± SEMs in the 30 olives (*n* = 6) and 60 olives (*n* = 7) groups.

The concentration–time profile of hydroxytyrosol was quantified for 8 h in both doses (Figure 2). Peak plasma concentrations (*C*_max_) of 23.44 ± 1.68 and 42.97 ± 1.39 nmol/L were achieved at 62 and 41 min for the doses of 30 and 60 olives, respectively (Table 1). However, a similar rate of absorption could be suggested between doses, according to the results obtained for the ratio *C*_max_/AUC, which was 0.0055 min^–1^ (30 olives) and 0.0048 min^–1^ (60 olives).

**Table 1:** Pharmacokinetic Parameters of Hydroxytyrosol Estimated by Noncompartmental Analysis^1^,^2^

		hydroxytyrosol	
parameters	units	30 olives group	60 olives group
amount administered	nmol	5272 ± 93	11195 ± 353
*C*_max_	nmol/L	23.44 (13.60, 40.38)^a^	42.97 (31.73, 58.18)^b^
*C*_max_/D		0.0044 (0.0026, 0.0077)^a^	0.0039 (0.0028, 0.0053)^a^
*T*_max_	min	62 (31, 91)^a^	41 (33, 68)^a^
AUC_t_	min·nmol/L	3363 (2642, 4280)a	7912 (6031, 10381)^b^
AUC_t_/D		0.6383 (0.5153, 0.7907)^a^	0.7091 (0.5407, 0.9300)^a^
AUC_0–∞_	min·nmol/L	4293 (3815, 4830)^a^	8919 (6920, 11495)^b^
AUC_0–∞_/D		0.8149 (0.7420, 0.8949)^a^	0.7993 (0.6170, 1.035)^a^
*C*_max_/AUC_0–∞_	1/min	0.0055 (0.0033, 0.0089)^a^	0.0048 (0.0038, 0.0062)^a^
λ_*z*_	1/min	0.0042 (0.0024, 0.0073)^a^	0.0063 (0.0046, 0.0088)^a^
*t*_1/2λ*z*_	min	166.2 (95.47, 289.2)^a^	109.7 (79.23, 152.0)^a^
MRT_0–∞_	min	255.5 (161.0, 405.6)^a^	206.1 (170.5, 249.1)^a^
Vss/F	L/kg	1139 (671.4, 1932)^a^	882.8 (674.2, 1161)^a^
Vd_area_/F	L/kg	1069 (580.0, 1969)^a^	678.3 (474.8, 969.0)^a^
Cl/F	L/min/kg	4.46 (3.96, 5.02)^a^	4.28 (3.32, 5.52)^a^

1 Plasma concentrations were obtained after a single oral administration to Sprague-Dawley rats of Arbequina table olives containing hydroxytyrosol at 2.95 mg/kg (30 olives group) and 5.89 mg/kg (60 olives group).

2 Results are expressed as means ± SEMs or geometric means (95% CIs) except for *T*_max_, which is presented as the median with its minimum and maximum value in the 30 olives (*n* = 6) and 60 olives (*n* = 7) groups. Data were analyzed by unpaired-Student *t*-test except for *T*_max_, in which a nonparametric Wilcoxon signed-rank test was used. Means without a common letter differ, *p* < 0.05. AUC_t_: area under the plasma concentration–time curve from time zero up to 8h; AUC_0–∞_, area under the plasma concentration–time curve from time zero to infinite; *C*_max_, peak plasma concentration; Cl/F, apparent clearance; D, dose of hydroxytyrosol; MRT_0–∞_, mean residence time from zero to infinite; *T*_max_, time to peak plasma concentration; *t*_1/2λz_, apparent elimination half-life; Vss/F, apparent steady-state volume of distribution; Vd_area_/F, apparent volume of distribution associated with the terminal phase of the plasma concentration–time curve; λ_*z*_, apparent elimination rate constant.

The exposure metrics of hydroxytyrosol (AUC_t_; AUC_0–∞_) increased significantly with dose (*p* < 0.05) in a proportional manner. This trend was confirmed by the lack of statistically significant differences when these variables were normalized by dose (Table 1). In addition, the plasma concentrations, normalized by dose versus time, plotted in log-scale showed an overlay of the curves (Figure 2). Overall, all of these data suggest a linear PK behavior for hydroxytyrosol under our experimental conditions.

The parameters characterizing the mean residence time (MRT_0–∞_) and the elimination of hydroxytyrosol from the body (λ_*z*_; *t*_1/2λ*z*_) are displayed in Table 1. The λ_*z*_ values were 0.0042/min (30 olives) and 0.0063/min (60 olives) resulting in half-life values (*t*_1/2λ*z*_) ranging from 2 to 3 h. These results indicated that about 10−15 h are required for the disappearance of the 97% of hydroxytyrosol from the body. This is in agreement with the MRT_0–∞_ which was approximately 4 h after the intake of 30 or 60 olives.

The apparent volumes of distribution at the steady state (*V*_ss_/*F*) (Table 1) were 1139.05 L/kg in the group of 30 olives and 882.82 L/kg in the 60 olives group, similar to Vd_area_/*F* values of 1069 and 678.3 L/kg for the groups of 30 and 60 olives, respectively. The values of apparent clearance (Cl/*F*) were also similar between the assayed doses, i.e., 4.46 (30 olives) and 4.28 L/min/kg (60 olives). The lack of statistically significant differences between doses for any of the pharmacokinetic parameters evaluated (λ_*z*_; *t*_1/2λ*z*_; MRT_0–∞_; V_ss_/F; Vd_area_/F) confirms the kinetic linearity for hydroxytyrosol over the studied dosage range.

#### 3.3.2 Metabolites of Hydroxytyrosol

The metabolites of hydroxytyrosol were quantified in plasma at all sampling times, the concentrations achieved by the two sulfates (M1-a and M1-b) being higher than the parent compound, while the two glucuronides (M2-a and M2-b) showed a lower exposure than hydroxytyrosol (Figure 2).

##### 3.3.2.1 Hydroxytyrosol Sulfates

The formation of sulfates M1-a and M1-b resulted in peak concentrations achieved at *T*_max_ values ranging from 30 to 45 min for both metabolites after the intake of the two doses (Table 2). The *C*_max_ for M1-b was 13 times higher than that of hydroxytyrosol for the two doses, whereas the sulfate M1-a was only double that of the parent compound. The highest AUC_t_ and AUC_0–∞_ were achieved by sulfate M1-b (Table 2). Within the same sulfate metabolites, the exposure parameters (AUC_t_; AUC_0–∞_) increased from the 30 to the 60 olives groups (*p* < 0.05), this increase being proportional because no statistically significant differences were found when they were normalized by dose (*p* > 0.05) (Table 2).

**Table 2:** Pharmacokinetic Parameters of the Metabolites of Hydroxytyrosol Estimated by Noncompartmental Analysis^1^,^2^

		hydroxytyrosol sulfate M1-a		hydroxytyrosol sulfate M1-b	
parameters	units	30 olives group	60 olives group	30 olives group	60 olives group
*C*_max_	(nmol/L)	47.34 (27.47, 81.60)^a^	97.35 (64.97, 145.9)^b^	306.4 (182.2, 515.3)^a^	548.4 (382.3, 786.5)^b^
*C*_max_/D		0.00899 (0.00522, 0.01546)^a^	0.00872 (0.00562, 0.01354)^a^	0.0582 (0.0343, 0.0985)^a^	0.0491 (0.0333, 0.0726)^a^
*T*_max_	min	45.5 (30, 120)^a^	36 (30, 42)^a^	30.50 (30,120)^a^	38 (30, 100)^a^
AUC_t_	min·nmol/L	4321 (3152, 5924)^a^	11376 (8674, 14919)^b^	26907 (19824, 36520)^a^	67548 (53591, 85140)^b^
AUC_t_/D		0.82 (0.60, 1.11)^a^	1.02 (0.75, 1.38)^a^	5.11 (3.78, 6.90)^a^	6.05 (4.76, 7.70)^a^
AUC_0–∞_	min·nmol/L	4765 (3491, 6504)^a^	13133 (9812, 17577)^b^	28084 (20399, 38664)^a^	80006 (65787, 97299)^b^
AUC_0–∞_/D		0.90 (0.68, 1.23)^a^	1.18 (0.84, 1.64)^a^	5.33 (3.89, 7.31)^a^	7.17 (5.62, 9.14)^a^
AUC_0–∞_m/AUC_0–∞_		1.11 (0.81, 1.52)^a^	1.47 (0.88, 2.46)^a^	6.54 (4.82, 8.8)^a^	8.97 (5.97, 13.47)^a^
λ_*z*_	(1/min)	0.0053 (0.0026, 0.011)^a^	0.0054 (0.0034, 0.0083)^a^	0.0080 (0.0063, 0.01024)^a^	0.0054 (0.0031, 0.0094)^a^
*t*_1/2λ*z*_	min	130.1 (64.5, 262.2)^a^	129.4 (83.0, 201.6)^a^	86.4 (67.7, 110)^a^	129.3 (74.1, 225.6)^a^
MRT_0–∞_	min	173 (121.9, 245.5)^a^	188.3 (143.6, 246,9)^a^	141.4 (118.1, 169.3)^a^	198.8 (139.0, 284.3)^a^
Vss/F·fm	L/kg	616 (339, 1120)^a^	548 (380, 789)^a^	96.4 (62.6, 148)^a^	83.5 (61.6, 112.9)^a^
Vd_area_/F·fm	L/kg	491 (190, 1264)^a^	479 (290, 790)^a^	85.0 (57.2, 126)^a^	75.1 (45.7, 123)^a^
Cl/F·fm	L/min/kg	4.02 (2.95, 5.48)^a^	2.91 (2.17, 3,89)^a^	0.68 (0.49, 0.94)^a^	0.48 (0.39, 0.58)^a^

		hydroxytyrosol glucuronide M2-a		hydroxytyrosol glucuronide M2-b	
parameters	units	30 olives group	60 olives group	30 olives group	60 olives group
*C*_max_	(nmol/L)	9.46 (5.99, 14.95)^a^	14.19 (9.34, 21.46)^a^	5.41 (3.30, 8.89)^a^	8.97 (6.16, 13.06)^a^
*C*_max_/D		0.00180 (0.00114, 0.00283)^a^	0.00127 (0.00081, 0.00201)^a^	0.00103 (0.0006, 0.0017)^a^	0.00080 (0.00052, 0.00124)^a^
*T*_max_	min	45.5 (30, 120)^a^	41 (30, 123)^a^	45.5 (30, 120)^a^	38 (30, 91)^a^
AUC_t_	min·nmol/L	1017 (845, 1224)^a^	1953 (1617, 2359)^b^	615.9 (475, 799)^a^	1161 (912.4, 1476)^b^
AUC_t_/D		0.19 (0.16, 0.23)^a^	0.18 (0.14, 0.22)^a^	0.12 (0.09, 0.15)^a^	0.10 (0.07, 0.14)^a^
AUC_0–∞_	min·nmol/L	1059 (862, 1301)^a^	2234 (1882, 2652)^b^	641.9 (491.1, 839.0)^a^	1239 (939.4, 1635)^b^
AUC_0–∞_/D		0.20 (0.17, 0.24)^a^	0.20 (0.16, 0.25)^a^	0.12 (0.09, 0.16)^a^	0.11 (0.079, 0.156)^a^
AUC_0–∞_m/AUC_0–∞_		0.25 (0.20, 0.30)^a^	0.25 (0.18, 0.35)^a^	0.15 (0.12, 0.19)^a^	0.14 (0.08, 0.22)^a^
λ_*z*_	(1/min)	0.0080 (0.0051, 0.0124)^a^	0.0062 (0.0039, 0.0010)^a^	0.0082 (0.0051, 0.0131)^a^	0.0078 (0.0050, 0.0122)^a^
*t*_1/2λ*z*_	min	86.92 (55.62, 135.9)^a^	111.8 (69.6, 179.6)^a^	84.84 (52.83, 136.2)^a^	88.58 (57.00, 137.7)^a^
MRT_0–∞_	min	142.8 (120.9, 168.7)^a^	185.6 (132.9, 259.2)^a^	138.8 (109.5, 17661)^a^	149.2 (123.1, 180.7)^a^
Vss/F·fm	L/kg	2580 (1950, 3414)^a^	2791 (2084, 3721)^a^	4139 (2668, 6420)^a^	4598 (3507, 6029)^a^
Vd_area_/F·fm	L/kg	2266 (1435, 3579)^a^	2386 (1497, 3803)^a^	3649 (2081, 6396)^a^	3940 (2433, 6380)^a^
Cl/F·fm	L/min/kg	18.1 (14.7, 22.2)^a^	17.1 (14.4, 20,3)^a^	29.8 (22.8, 38.9)^a^	30.8 (23.4, 40.7)^a^

1 Plasma concentrations were obtained after a single oral administration to Sprague-Dawley rats of Arbequina table olives containing hydroxytyrosol at 2.95 mg/kg (30 olives group) and 5.89 mg/kg (60 olives group).

2 Results are expressed as geometric means (95% CIs) except for *T*_max_ which is presented as the median with its minimum and maximum value in the 30 olives (*n* = 6) and 60 olives (*n* = 7) groups. Data were analyzed by unpaired-Student *t*-test except for *T*_max_ in which a nonparametric Wilcoxon signed-rank test was used. Within the same compound, means without a common letter differ, *p* < 0.05. AUC_0-∞_, area under the plasma concentration–time curve from time zero to infinite; AUC_t_: area under the plasma concentration–time curve from time zero up to 8h; AUC_0-∞_m/AUC_0-∞_, ratio of the relative exposure of each metabolite with respect to hydroxytyrosol; *C*_max_, peak plasma concentration; Cl/F·fm, apparent clearance of metabolite; D, dose of hydroxytyrosol; MRT_0-∞_, mean residence time from zero to infinite; *T*_max_, time to peak plasma concentration; *t*_1/2λz,_ apparent elimination half-life; V_ss_/F·fm, apparent steady-state volume of distribution of metabolite; Vd_area_/F·fm, apparent volume of distribution associated with the terminal phase of the plasma concentration–time curve of metabolite; λ_*z*_, apparent elimination rate constant.

Concerning the elimination phase, the λ_*z*_ values were not significantly different from those of hydroxytyrosol for both M1-a and M1-b metabolites. The *t*_1/2λ*z*_ was approximately 2 h for M1-a and M1-b at the two doses, while the average time that these metabolites spent in the body (MRT_0–∞_) was found to be around 3 h (Table 2). The apparent volumes of distribution were different for the two derivatives (Table 2). In this sense, the estimated V_ss_/F·fm and Vd_area_/F·fm for the sulfate M1-a were approximately 500 L/kg, whereas for the sulfate M1-b they were close to 90 L/kg. Differences in both parameters between the two groups of 30 and 60 olives were not relevant, this confirming again the linear pharmacokinetic behavior between both doses. A higher apparent clearance was observed for the sulfate M1-a compared to the M1-b, without statistically significant differences between doses. Hence, the metabolite M1-a had a Cl/F·fm of 4.02 L/min/kg (30 olives) and 2.91 L/min/kg (60 olives), whereas the most abundant sulfate derivative (M1-b) exhibited a Cl/F·fm of 0.68 L/min/kg (30 olives) and 0.48 L/min/kg (60 olives).

##### 3.3.2.2 Hydroxytyrosol Glucuronides

The analysis of the peak plasma concentrations of the glucuronide metabolites led to a *T*_max_ of 45 min for M2-a and M2-b at the two doses (Table 2). The *C*_max_ of M2-a and M2-b were 0.3 and 0.2 times lower than hydroxytyrosol in the two groups (Table 2). When the exposure parameters were compared between doses, the AUC from the 60 olives group was significantly higher than the AUC in the 30 olives group (*p* < 0.05). However, no statistically significant differences were found when these parameters were normalized by dose (Table 2).

The PK parameters describing the elimination phase of the glucuronides were the shortest for both metabolites as stated by a λ_*z*_ of approximately 0.008/min, a *t*_1/2 λ*z*_ of around 1.5 h, and MRT_0–∞_ of 2.5 h (Table 2). The apparent volumes of distribution for the glucuronides were higher than those estimated for hydroxytyrosol or the sulfate derivatives (Table 2). The glucuronide M2-a yielded V_ss_/F·fm and Vd_area_/F·fm of around 2500 L/kg, while the glucuronide M2-b was approximately 4000 L/kg, without differences between doses. Regarding apparent clearance, the glucuronide metabolites had higher values than hydroxytyrosol and hydroxytyrosol sulfate. The M2-a metabolite had a value of Cl/F·fm of 18.1 L/min/kg (30 olives) and 17.1 L/min/kg (60 olives), whereas the Cl/F·fm of M2-b were 29.8 L/min/kg (30 olives) and 30.8 L/min/kg (60 olives).

#### 3.3.3 Comparison of the Pharmacokinetic Behavior of Hydroxytyrosol versus Its Metabolites

**Figure 3:**
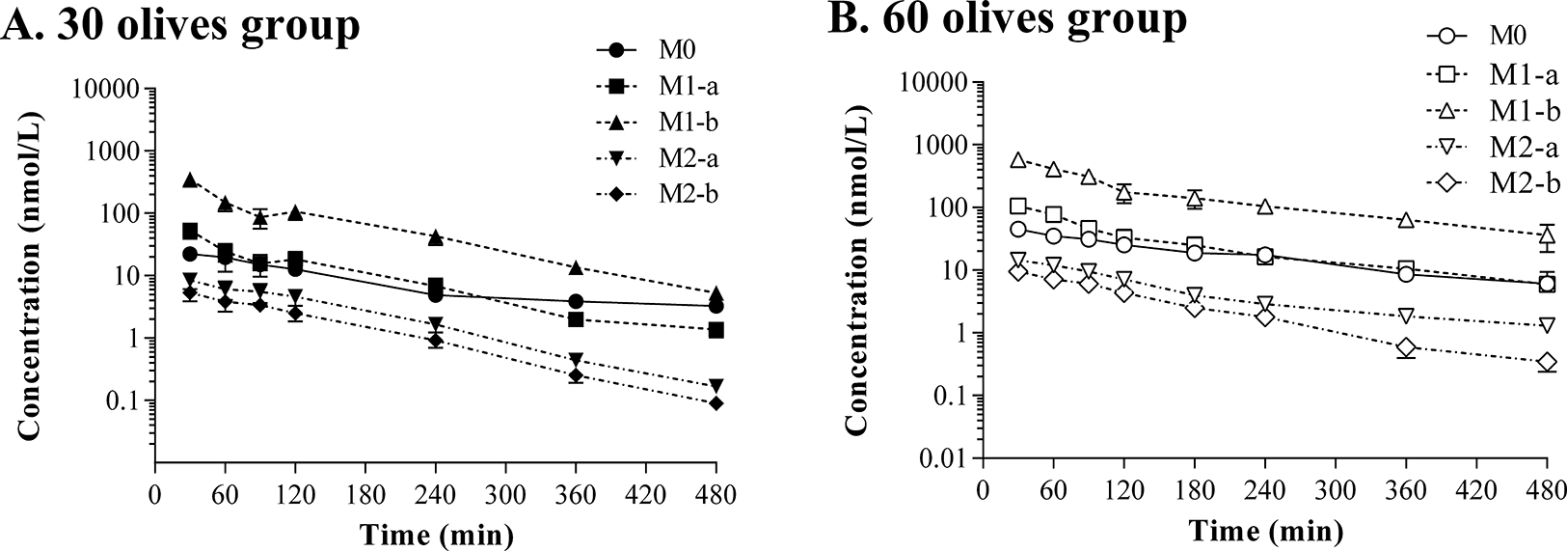
Plasma concentrations plotted in a semilogarithmic scale of hydroxytyrosol (M0), hydroxytyrosol sulfates (M1-a, M1-b), and hydroxytyrosol glucuronides (M2-a, M2-b) obtained for the doses of hydroxytyrosol of 2.95 mg/kg and 5.89 mg/kg administered to Sprague-Dawley in the 30 olives group (A) and 60 olives group (B). Results are expressed as means ± SEMs in the 30 olives (*n* = 6) and 60 olives (*n* = 7) groups.

The overlay of the concentrations of hydroxytyrosol and its metabolites plotted in a semilogarithmic scale versus time displayed a parallel one-exponential decay for all the compounds at both doses (Figure 3). No statistically significant differences were found for the slopes between compounds and doses.

The ratio of the AUC for each metabolite to the AUC of hydroxytyrosol (AUC_0–∞_m/AUC_0–∞_) was calculated to get an insight into the quantitative significance of each derivative with respect to the parent compound (Table 2). Sulfate M1-b showed the highest value that ranged from 7 to 9, followed by the sulfate M1-a at around 1.5. A marked decrease in this ratio was observed for the glucuronides, being 0.25 for metabolite M2-a and 0.15 for M2-b.

**Figure 4:**
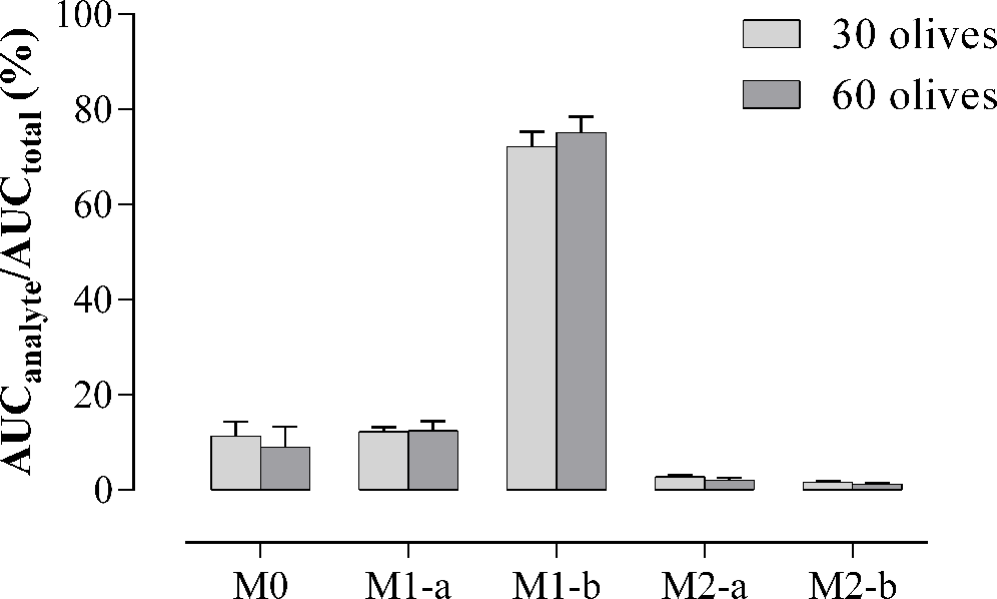
Ratio of the area under the curve (AUC) of hydroxytyrosol (M0), hydroxytyrosol sulfates (M1-a, M1-b), and hydroxytyrosol glucuronides (M2-a, M2-b) with respect to the total AUC expressed in percentage obtained after the oral administration of 2.95 mg/kg (30 olives group) and 5.89 mg/kg (60 olives group) to Sprague-Dawley. Results are represented as means ± SEMs in the 30 olives (*n* = 6) and 60 olives (*n* = 7) groups.

The extent of the metabolism undergone by hydroxytyrosol was also assessed by calculating the percentage of the AUC of each compound divided by the AUC of the sum of all of them. Figure 4 shows that similar ratios were obtained between doses (*p* > 0.05). The analysis of the AUC of hydroxytyrosol and its derivatives indicated that the sulfate M1-b accounted for approximately 73% of all the compounds, followed by the sulfate M1-a (12%), hydroxytyrosol (11%), and the two glucuronides with values around 2% for each metabolite.

## 4 Discussion

In the present study, the PK of hydroxytyrosol as well as its sulfate and glucuronide metabolites have been characterized in the plasma of Sprague-Dawley rats after the oral intake of Arbequina table olives. To this aim, hydroxytyrosol was determined using previously established methodologies by LC-MS/MS in Arbequina table olives^9^ and rat plasma.^11^ The validation of the methods in both olives and plasma provided LOQ values of 3 and 0.2 nM, respectively. These sensitivities ensured the accurate measurement of hydroxytyrosol even at low concentrations, which overcomes one of the drawbacks of previous studies mainly in plasma.^34^ Noteworthy, the method enabled the chromatographic separation of hydroxytyrosol from its phase II derivatives and the quantification of not only the parent compound but also its sulfate and glucuronide forms, unlike other methods that used enzymatic hydrolysis.^19,21,22^ Moreover, our extraction process required a small volume of plasma, allowing all samples to be obtained from the same animal, not like other studies that used two rats per sampling time^19^ or only three sampling times per rat, according to a sparse sampling designs.^23^ The advantages are that because of the analytical method, mean values of PK parameters were obtained from the individual PK parameter values estimated for each rat. Consequently, unlike the sparse sampling designs, in which mean PK parameters are estimated from pooled data of different animals, interindividual variability does not affect the mean values. This contributes to the estimation of more precise PK parameter values, with a lower sample size, a fact of great importance from an ethical and cost point of view.^35^

The methodology used in the current study allowed the quantification of hydroxytyrosol and its sulfate and glucuronide metabolites for up to 8 h, in contrast to previous research, often reporting values at few extraction times or presenting total concentrations after hydrolysis without specific information on metabolites.^18−23^ Some studies, such as Bai et al.^18^ only described plasmatic concentrations up to 3 h after the administration of 10 mg/mL of this polyphenol without giving data on metabolites. Similarly, Serra et al.^23^ collected blood at 1, 2, and 4 h postadministration of a phenolic extract from olive cake containing 10.35 mg/kg of hydroxytyrosol, while López de las Hazas et al.^20^ obtained blood at 5 h following the oral intake of hydroxytyrosol at 1, 10, and 100 mg/kg. Other research faces limitations not only due to reporting concentrations in few sampling points but also because plasma samples were processed by enzymatic hydrolysis, resulting in reported concentrations of hydroxytyrosol together with its conjugates.^19,21,22^ In these studies, Domínguez-Perles et al.^21^ could determine hydroxytyrosol up to 2 h following oral administration of doses of 1 and 5 mg/kg, while Kano et al.^22^ detected hydroxytyrosol up to 4 h after gavage of 100 mg/kg. D’Angelo et al.^19^ reported plasma concentrations up to 5 h after intravenous administration of a dose of 1.5 mg/kg radiolabeled hydroxytyrosol.

Given the limited pharmacokinetic data from previous studies, our findings provide relevant insights to properly characterize the PK behavior. After oral administration of the doses of 3.85 and 7.70 g of destoned olives/kg that supplied hydroxytyrosol at 2.95 and 5.89 mg/kg, we quantified the parent compound along with its sulfate and glucuronide conjugates in plasma at 7 sampling times ranging from 30 min to 8 h postdose. Noncompartmental analysis of the plasma concentration–time profiles showed that hydroxytyrosol was absorbed from olives with a *T*_max_ of approximately 60 and 40 min for the 30 and 60 olives doses, respectively. Caution should be taken when interpreting these results, given that *T*_max_ values are obtained from one single point and are highly influenced by the sampling scheme, resulting in less precise estimates than *C*_max_/AUC in which estimation involves more observed data. Then, observed *T*_max_ differences should not be considered as indicative of different absorption rates.^36^ These *T*_max_ values are longer than data in the literature that come mainly from studies performed using the pure compound, devoid of the complex matrix environment of table olives. In such cases, where hydroxytyrosol is readily available for absorption, a shorter *T*_max_ of 5–10 min^18^ was described. However, when hydroxytyrosol was quantified after enzymatic hydrolysis, the *T*_max_ was 15 min^22^ or between 0.5 and 1 h.^21^ The longest *T*_max_ of 2 h was reported by Serra et al.,^23^ although their sampling times were limited to 1, 2, and 4 h. Overall, our findings indicate that hydroxytyrosol absorption from olives is somewhat delayed compared with that of the pure compound, likely reflecting release from the food matrix. Indeed, assuming a first-order absorption kinetic process, and taking into account the estimated *C*_max_/AUC values of 0.0055–0.0048 min^–1^, absorption half-life values around 126–144 min can be inferred, as well as total absorption periods of 21–24 h. These periods of time are close to the times required for the total disappearance of hydroxytyrosol from the body (around 10 half-life values, i.e., 27.7–18.3 h), this fact suggesting a delayed absorption of hydroxytyrosol from table olives.

The assessment of dose linearity was considered an important issue in elucidating the PK of hydroxytyrosol following the intake of table olives, because this knowledge could facilitate the prediction of the concentrations of this polyphenol at different dosage regimens and guide evidence-based dietary recommendations of the consumption of this food. Analysis of plasma concentrations revealed a proportional increase in hydroxytyrosol, as evidenced by the doubling of *C*_max_, AUC_t_, and AUC_0-∞_ at the higher dose of 7.70 compared to the lower dose of 3.85 g of destoned olives/kg. Furthermore, the linear PK behavior was verified when these parameters were normalized by dose, indicating no statistically significant differences between doses. Our results agree with those found by López de las Hazas et al.^20^ who reported a proportional increase in the plasma concentrations of hydroxytyrosol obtained 5 h after the administration of 1, 10, and 100 mg/kg dissolved in refined olive oil. However, Domínguez-Perles et al.^21^ did not observe linear PK after the administration of 1 and 5 mg/kg of hydroxytyrosol, probably because they measured the total concentrations in plasma of the parent compound and metabolites obtained after enzymatic hydrolysis.

Given that we did not have data from the intravenous administration of hydroxytyrosol, the apparent distribution volumes were unidentifiable. However, a possible approximation to the distribution volume of hydroxytyrosol could be estimated by the ratio V/F. The values of either V_ss_/F or Vd_area_/F obtained in our study were approximately 1000 L/kg for both doses, suggesting an extensive distribution rather than being restricted to plasma. These values exceed the typical total body water volume in an adult rat (around 0.7 L/kg), thus being indicative of an extensive distribution into tissues.^37^ Our results agreed with previous findings in rats that describe the distribution of hydroxytyrosol, particularly in highly perfused organs such as the kidney and liver.^19,20,23^ Additionally, an accumulation of hydroxytyrosol has been observed in the testes^23^ and erythrocytes.^38^ Finally, lower concentrations were detected in the brain, heart, lung, and skeletal muscle.^19,20^ The absence of intravenous data hampers the estimation of hydroxytyrosol clearance, but the estimation of Cl/F was around 4.3 L/min, that although being an apparent value is relatively high compared to liver blood flow (1.4 L/min in rats).^37^ Then, the interpretation of Cl/F could be due to either a high extraction rate, low bioavailability, or both of them. Clearance values observed in our study are consistent with the extensive metabolism found for hydroxytyrosol, as demonstrated by the circulating plasma metabolites consisting of 89% sulfate and glucuronide conjugates versus only 11% unmodified hydroxytyrosol. On the other hand, the PK of hydroxytyrosol leads to a *t*_1/2λz_ of approximately 2.5 h, and the MRT_0–∞_ of around 4 h is slightly longer than the *t*_1/2λ*z*_, suggesting that hydroxytyrosol has a moderate persistence in the body, that could be explained by distribution into peripheral organs as implied by the high apparent volumes of distribution.

Regarding the PK of the metabolites, the *T*_max_ ranged from 30 to 45 min, which is a short time compared to the half-life values for metabolites ranging from 86 to 130 min. Moreover, the semilogarithmic concentration–time profiles of hydroxytyrosol and its derivatives show a parallel decline of the terminal phase of all the metabolites with respect to the parent compound. This was confirmed by the lack of statistically significant differences between the respective slopes of the terminal phase, which suggests that the elimination of the metabolites is limited by their formation. The analysis of the AUC_0–∞_m of the metabolites entails considering several PK factors beyond those influencing the parent compound. While the AUC_0–∞_ of hydroxytyrosol depends on the oral dose, the fraction absorbed, and the clearance, the AUC_0–∞_m of the metabolites relies additionally on the fraction of hydroxytyrosol converted to that conjugate (fm) and their specific clearances. Consequently, the systemic exposure of the conjugates could be better assessed by calculating the ratio of metabolite AUC_0–∞_m to parent AUC_0–∞_. Normalizing the estimated metabolite exposure to that of the parent compound also provides valuable insight into whether the metabolite will be quantitatively important *in vivo*. These ratios are independent of the dose and fraction of the absorbed parent compound, so they could be used to predict the values of AUC_0–∞_m when a specific AUC is required (AUC_0–∞_m/AUC_0–∞_ = fm·Cl/Clm). The much higher ratio for sulfate M1-b around 7–9-fold indicates sulfation as the major pathway compared to glucuronidation, with ratios below 0.25. Our results agree with previous studies in rats indicating that this polyphenol undergoes predominantly phase II processes, with a predominance of the sulfation pathway.^23,20,39^ Similarly to our study, Serra et al.^23^ reported in rat plasma a 92% of hydroxytyrosol sulfate, a 6% of free polyphenol, and a 2% of hydroxytyrosol glucuronide after the administration of a phenolic extract from olive cake containing 10.35 mg/kg of hydroxytyrosol. López de las Hazas et al.^20^ also indicated that sulfation was the most relevant conjugation pathway compared to the glucuronide conjugate after the supplementation with hydroxytyrosol at 1, 10, and 100 mg/kg. Consistent with those findings, Kotronoulas et al.^39^ reported that hydroxytyrosol sulfates represented a 60 and 75% after the oral administration of 10 and 100 mg/kg of hydroxytyrosol to rats. In addition to these derivatives, hydroxytyrosol also undergoes modifications to other metabolites involved in dopamine biotransformation, such as 3,4-dihydroxyphenylacetic acid (DOPAC), homovanillic acid (HVA), and their glucuronidated and sulfate metabolites, although in lower amounts.^34−40^ Therefore, in our study, we focused on phase II derivatives because despite being described as predominant hydroxytyrosol metabolites, the majority of research in rats^19,21,22^ and humans^12,14,25^ reported plasma concentrations only after enzymatic hydrolysis. Thus, most investigations, unlike our study, cannot discriminate the actual systemic concentrations of the parent compound from those of its sulfate and glucuronide conjugates.

Although there were no prior pharmacokinetic data available in the literature characterizing the PK of hydroxytyrosol in rats after the intake of table olives, the plasma profile has been previously described in healthy volunteers after the intake of Kalamata olives.^25,26^ Goldberg et al.^26^ administrated 10 olives (assuming a mass of a single olive of 25 g) with 389.87 mg hydroxytyrosol/kg, while Kountouri et al.^25^ reported a consumption of 20 olives (approximately 100 g) containing 767.3 mg hydroxytyrosol/kg. In terms of plasma concentrations, Goldberg et al.^26^ gave the results of the parent compound with a *C*_max_ of 0.64 pmol/mL (0.65 nmol/L) obtained at 30 min, reporting that exposure was very small because the AUC was 39 pmol·min/mL (0.65 nmol·h/L), which is much inferior to the one reported in our study. On the other hand, Kountouri et al.^25^ described a *C*_max_ of 3.145 μg/mL (20.4 μmol/L) at 1 h. However, those values were obtained after enzymatic hydrolysis without providing data on free hydroxytyrosol. Our results are most in line with the ones obtained in humans after the intake of oil, even though no free hydroxytyrosol was determined, and the plasma concentrations were obtained after enzymatic hydrolysis.^12,14^ Following oral intake of 5 mg of hydroxytyrosol in EVOO by humans, Alemán-Jiménez et al.^14^ described a *C*_max_ of 3.79 ng/mL (24.6 nmol/L) at 30 min, and the values obtained after 1 h up to 4 h were not different from the controls. Similarly, Miro-Casas et al.^12^ reported *C*_max_ of approximately 130 nmol/L at 30 min after the consumption of 25 mL of virgin olive in healthy human volunteers. Hence our results provide a novelty from the existing data because we could determine plasma concentrations of free hydroxytyrosol from 30 min to 8 h, with *C*_max_ for the parent compound of 23.44 and 42.97 nmol/L. Taking into account all the metabolites, our *C*_max_ values were 393.05 nm/L (30 olives group) and 747.88 nmol/L (60 olives group), which are higher than the ones reported after the intake of olive oil. These results emphasize the potential implications of the matrix used for administering this polyphenol, on its absorption kinetics and duration of hydroxytyrosol in the systemic circulation.

In summary, our results showed that unlike other matrices, when given in table olives, hydroxytyrosol shows a delayed absorption that in turn provides sustained plasma concentrations. This study may be the first stage toward future research to address the optimal exposure and doses to obtain beneficial effects. All of these results will be useful to consider table olives as a promising functional food.

## Supporting Information Available

Chemical structures of hydroxytyrosol and its sulfate and glucuronide conjugates (PDF)

## References

[ref1] Maruca A.; Catalano R.; Bagetta D.; Mesiti F.; Ambrosio F. A.; Romeo I.; Moraca F.; Rocca R.; Ortuso F.; Artese A.; Costa G.; Alcaro S.; Lupia A. The Mediterranean Diet as source of bioactive compounds with multi-targeting anti-cancer profile. Eur. J. Med. Chem. 2019, 181, 111579 10.1016/j.ejmech.2019.111579. 31398616

[ref2] Juan M. E.; Wenzel U.; Daniel H.; Planas J. M. Cancer chemopreventive activity of hydroxytyrosol: a natural antioxidant from olives and olive oil. In Olives and Olive Oil in Health and Disease Prevention; Preedy V. R., Watson R. R., Eds.; Academic Press, Elsevier: Cambridge, MA, 2010; pp 1295–1300.

[ref3] Karković Marković A.; Torić J.; Barbarić M.; Jakobušić Brala C. Hydroxytyrosol, tyrosol and derivatives and their potential effects on human health. Molecules 2019, 24 (10), 2001 10.3390/molecules24102001. 31137753 PMC6571782

[ref4] Bertelli M.; Kiani A. K.; Paolacci S.; Manara E.; Kurti D.; Dhuli K.; Bushati V.; Miertus J.; Pangallo D.; Baglivo M.; Beccari T.; Michelini S. Hydroxytyrosol: A natural compound with promising pharmacological activities. J. Biotechnol. 2020, 309, 29–33. 10.1016/j.jbiotec.2019.12.016. 31884046

[ref5] Terracina S.; Petrella C.; Francati S.; Lucarelli M.; Barbato C.; Minni A.; Ralli M.; Greco A.; Tarani L.; Fiore M.; Ferraguti G. Antioxidant intervention to improve cognition in the aging brain: the example of hydroxytyrosol and resveratrol. Int. J. Mol. Sci. 2022, 23 (24), 15674 10.3390/ijms232415674. 36555317 PMC9778814

[ref6] Noguera-Navarro C.; Montoro-García S.; Orenes-Piñero E. Hydroxytyrosol: Its role in the prevention of cardiovascular diseases. Heliyon 2023, 9 (1), e12963 10.1016/j.heliyon.2023.e12963. 36704293 PMC9871206

[ref7] Commission Regulation (EU) No 432/2012 of 16 May 2012 establishing a list of permitted health claims made on foods, other than those referring to the reduction of disease risk and to children’s development and health. Off. J. Eur. Union 2012, 136, 1.

[ref8] Gallardo-Fernández M.; Gonzalez-Ramirez M.; Cerezo A. B.; Troncoso A. M.; Garcia-Parrilla M. C. Hydroxytyrosol in foods: analysis, food sources, EU dietary intake, and potential uses. Foods 2022, 11 (15), 2355 10.3390/foods11152355. 35954121 PMC9368174

[ref9] Moreno-González R.; Juan M. E.; Planas J. M. Table olive polyphenols: A simultaneous determination by liquid chromatography-mass spectrometry. J. Chromatogr. A 2020, 1609, 460434 10.1016/j.chroma.2019.460434. 31416621

[ref10] Moreno-González R.; Juan M. E.; Planas J. M. Profiling of pentacyclic triterpenes and 435 polyphenols by LC-MS in Arbequina and Empeltre table olives. LWT-Food Sci. Technol. 2020, 126, 109310 10.1016/j.lwt.2020.109310.

[ref11] Kundisová I.; Juan M. E.; Planas J. M. Simultaneous Determination of phenolic compounds in plasma by lc-esi-ms/ms and their bioavailability after the ingestion of table olives. J. Agric. Food Chem. 2020, 68 (37), 10213–10222. 10.1021/acs.jafc.0c04036. 32833444

[ref12] Miro-Casas E.; Covas M. I.; Farre M.; Fito M.; Ortuño J.; Weinbrenner T.; Roset P.; de la Torre R. Hydroxytyrosol disposition in humans. Clin. Chem. 2003, 49 (6), 945–952. 10.1373/49.6.945. 12765992

[ref13] Silva S.; Garcia-Aloy M.; Figueira M. E.; Combet E.; Mullen W.; Bronze M. R. High-resolution mass spectrometric analysis of secoiridoids and metabolites as biomarkers of acute olive oil intake-an approach to study interindividual variability in humans. Mol. Nutr. Food Res. 2018, 62, 1700065 10.1002/mnfr.201700065. 29068138

[ref14] Alemán-Jiménez C.; Domínguez-Perles R.; Medina S.; Prgomet I.; López-González I.; Simonelli-Muñoz A.; Campillo-Cano M.; Auñón D.; Ferreres F.; Gil-Izquierdo Á. Pharmacokinetics and bioavailability of hydroxytyrosol are dependent on the food matrix in humans. Eur. J. Nutr. 2021, 60 (2), 905–915. 10.1007/s00394-020-02295-0. 32524230

[ref15] de Bock M.; Thorstensen E. B.; Derraik J. G.; Henderson H. V.; Hofman P. L.; Cutfield W. S. Human absorption and metabolism of oleuropein and hydroxytyrosol ingested as olive (*Olea europaea* L.) leaf extract. Mol. Nutr. Food Res. 2013, 57 (11), 2079–2085. 10.1002/mnfr.201200795. 23766098

[ref16] García-Villalba R.; Larrosa M.; Possemiers S.; Tomás-Barberán F. A.; Espín J. C. Bioavailability of phenolics from an oleuropein-rich olive (*Olea europaea* L.) leaf extract and its acute effect on plasma antioxidant status: comparison between pre- and postmenopausal women. Eur. J. Nutr. 2014, 53 (4), 1015–1027. 10.1007/s00394-013-0604-9. 24158653

[ref17] González-Santiago M.; Fonollá J.; Lopez-Huertas E. Human absorption of a supplement containing purified hydroxytyrosol, a natural antioxidant from olive oil, and evidence for its transient association with low-density lipoproteins. Pharmacol. Res. 2010, 61 (4), 364–370. 10.1016/j.phrs.2009.12.016. 20045462

[ref18] Bai C.; Yan X.; Takenaka M.; Sekiya K.; Nagata T. Determination of synthetic hydroxytyrosol in rat plasma by GC-MS. J. Agric. Food Chem. 1998, 46 (10), 3998–4001. 10.1021/jf980451r.

[ref19] D’Angelo S.; Manna C.; Migliardi V.; Mazzoni O.; Morrica P.; Capasso G.; Pontoni G.; Galletti P.; Zappia V. Pharmacokinetics and metabolism of hydroxytyrosol, a natural antioxidant from olive oil. Drug Metab. Dispos. 2001, 29 (11), 1492–1498. 11602527

[ref20] López de las Hazas M. C.; Rubió L.; Kotronoulas A.; de la Torre R.; Solà R.; Motilva M. J. Dose effect on the uptake and accumulation of hydroxytyrosol and its metabolites in target tissues in rats. Mol. Nutr. Food Res. 2015, 59 (7), 1395–1399. 10.1002/mnfr.201500048. 25808038

[ref21] Domínguez-Perles R.; Auñón D.; Ferreres F.; Gil-Izquierdo A. Gender differences in plasma and urine metabolites from Sprague–Dawley rats after oral administration of normal and high doses of hydroxytyrosol, hydroxytyrosol acetate, and DOPAC. Eur. J. Nutr. 2017, 56 (1), 215–224. 10.1007/s00394-015-1071-2. 26463517

[ref22] Kano S.; Komada H.; Yonekura L.; Sato A.; Nishiwaki H.; Tamura H. Absorption, metabolism, and excretion by freely moving rats of 3,4-DHPEA-EDA and related polyphenols from olive fruits (Olea europaea L.). J. Nutr. Metab. 2016, 2016, 9104208 10.1155/2016/9104208. 26904279 PMC4745926

[ref23] Serra A.; Rubió L.; Borràs X.; Macià A.; Romero M. P.; Motilva M. J. Distribution of olive oil phenolic compounds in rat tissues after administration of a phenolic extract from olive cake. Mol. Nutr. Food Res. 2012, 56 (3), 486–496. 10.1002/mnfr.201100436. 22183818

[ref24] Rein M. J.; Renouf M.; Cruz-Hernandez C.; Actis-Goretta L.; Thakkar S. K.; da Silva Pinto M. Bioavailability of bioactive food compounds: a challenging journey to bioefficacy. Br. J. Clin. Pharmacol. 2013, 75 (3), 588–602. 10.1111/j.1365-2125.2012.04425.x. 22897361 PMC3575927

[ref25] Kountouri A. M.; Mylona A.; Kaliora A. C.; Andrikopoulos N. K. Bioavailability of the phenolic compounds of the fruits (drupes) of Olea europaea (olives): impact on plasma antioxidant status in humans. Phytomedicine 2007, 14 (10), 659–667. 10.1016/j.phymed.2007.06.001. 17870451

[ref26] Goldstein D. S.; Holmes C.; Cherup J.; Sharabi Y. Plasma catechols after eating olives. Clin. Transl. Sci. 2018, 11 (1), 32–37. 10.1111/cts.12489. 28898548 PMC5759722

[ref27] Serra-Majem L.; Tomaino L.; Dernini S.; Berry E. M.; Lairon D.; Ngo de la Cruz J.; Bach-Faig A.; Donini L. M.; Medina F. X.; Belahsen R.; Piscopo S.; Capone R.; Aranceta-Bartrina J.; La Vecchia C.; Trichopoulou A. Updating the mediterranean diet pyramid towards sustainability: focus on environmental concerns. Int. J. Environ. Res. Public Health 2020, 17 (23), 8758 10.3390/ijerph17238758. 33255721 PMC7728084

[ref28] Franco-Ávila T.; Moreno-González R.; Juan M. E.; Planas J. M. Table olive elicits antihypertensive activity in spontaneously hypertensive rats. J. Sci. Food Agric. 2023, 103 (1), 64–72. 10.1002/jsfa.12112. 35804485 PMC9796528

[ref29] Gómez-Contreras A.; Franco-Ávila T.; Miró L.; Juan M. E.; Moretó M.; Planas J. M. Dietary intake of table olives exerts antihypertensive effects in association with changes in gut microbiota in spontaneously hypertensive rats. Food Funct 2023, 14 (6), 2793–2806. 10.1039/D2FO02928F. 36861461

[ref30] Reagan-Shaw S.; Nihal M.; Ahmad N. Dose translation from animal to human studies revisited. FASEB J. 2008, 22 (3), 659–661. 10.1096/fj.07-9574LSF. 17942826

[ref31] Sánchez-González M.; Colom H.; Lozano-Mena G.; Juan M. E.; Planas J. M. Population pharmacokinetics of maslinic acid, a triterpene from olives, after intravenous and oral administration in rats. Mol. Nutr. Food Res. 2014, 58 (10), 1970–1979. 10.1002/mnfr.201400147. 25045029

[ref32] Mackie C.; Wuyts K.; Haseldonckx M.; Blokland S.; Gysemberg P.; Verhoeven I.; Timmerman P.; Nijsen M. New model for intravenous drug administration and blood sampling in the awake rat, designed to increase quality and throughput for *in vivo* pharmacokinetic analysis. J. Pharmacol. Toxicol. Methods 2005, 52 (2), 293–301. 10.1016/j.vascn.2004.11.002. 16125629

[ref33] Guideline on Bioanalytical Method Validation: Committee for Medicinal Products for Human Use (CHMP); European Medicines Agency (EMA): London, 2011.

[ref34] Rodríguez-Morató J.; Boronat A.; Kotronoulas A.; Pujadas M.; Pastor A.; Olesti E.; Pérez-Mañá C.; Khymenets O.; Fitó M.; Farré M.; de la Torre R. Metabolic disposition and biological significance of simple phenols of dietary origin: hydroxytyrosol and tyrosol. Drug Metab. Rev. 2016, 48 (2), 218–236. 10.1080/03602532.2016.1179754. 27186796

[ref35] Harstad E.; Andaya R.; Couch J.; Ding X.; Liang X.; Liederer B. M.; Messick K.; Nguyen T.; Schweiger M.; Tarrant J.; Zhong S.; Dean B. Balancing blood sample volume with 3Rs: implementation and best practices for small molecule toxicokinetic assessments in rats. ILAR J. 2016, 57 (2), 157–165. 10.1093/ilar/ilw023. 28053069

[ref36] Tothfalusi L.; Endrenyi L. Estimation of C_max_ and T_max_ in populations after single and multiple drug administrations. J. Pharmacokinet Pharmacodyn 2003, 30 (5), 363–385. 10.1023/B:JOPA.0000008159.97748.09. 14977165

[ref37] Davies B.; Morris T. Physiological parameters in laboratory animals and humans. Pharm. Res. 1993, 10 (7), 1093–1095. 10.1023/A:1018943613122. 8378254

[ref38] Rubió L.; Serra A.; Macià A.; Piñol C.; Romero M. P.; Motilva M. J. *In vivo* distribution and deconjugation of hydroxytyrosol phase II metabolites in red blood cells: a potential new target for hydroxytyrosol. J. Funct. Foods 2014, 10, 139–143. 10.1016/j.jff.2014.06.001.

[ref39] Kotronoulas A.; Pizarro N.; Serra A.; Robledo P.; Joglar J.; Rubió L.; Hernaéz A.; Tormos C.; Motilva M. J.; Fitó M.; Covas M. I.; Solà R.; Farré M.; Saez G.; de la Torre R. Dose-dependent metabolic disposition of hydroxytyrosol and formation of mercapturates in rats. Pharmacol. Res. 2013, 77, 47–56. 10.1016/j.phrs.2013.09.001. 24044986

[ref40] Galmés S.; Reynés B.; Palou M.; Palou-March A.; Palou A. Absorption, distribution, metabolism, and excretion of the main olive tree phenols and polyphenols: a literature review. J. Agric. Food Chem. 2021, 69 (18), 5281–5296. 10.1021/acs.jafc.1c00737. 33908772

