# Supplementary material for: Pharmacokinetics of Hydroxytyrosol and Its Sulfate and Glucuronide Metabolites after the Oral Administration of Table Olives to Sprague-Dawley Rats
Source: J Agric Food Chem. 2024 Jan 17;72(4):2154–64. doi: 10.1021/acs.jafc.3c06431 (PMC10835732; doi:10.1021/acs.jafc.3c06431)
Supplement: Supplementary file 1 — jf3c06431_si_001.pdf [file jf3c06431_si_001.pdf]

SUPPORTING INFORMATION

Pharmacokinetics of hydroxytyrosol and its sulfate and glucuronide metabolites after the oral administration of table olives to Sprague–Dawley rats

Ivana Kundisová[1], Helena Colom[2], M. Emília Juan[1] and Joana M. Planas[1]

[1]Grup de Fisiologia i Nutrició Experimental, Departament de Bioquímica i Fisiologia, Facultat de Farmàcia i Ciències de l'Alimentació and Institut de Recerca en Nutrició i Seguretat Alimentària (INSA-UB, Maria de Maeztu Unit of Excellence), Universitat de Barcelona (UB), and Food Innovation Network (XIA). Av. Joan XXIII 27-31, 08028-Barcelona, Spain.

[2]Grup de Farmacocinètica, Famacodinàmia i Farmacogenòmica poblacional, Departament de Farmàcia i Tecnologia Farmacèutica, i Fisicoquímica, Facultat de Farmàcia i Ciències de l'Alimentació, Universitat de Barcelona (UB). Av. Joan XXIII 27-31, 08028-Barcelona, Spain.

Supplementary Material

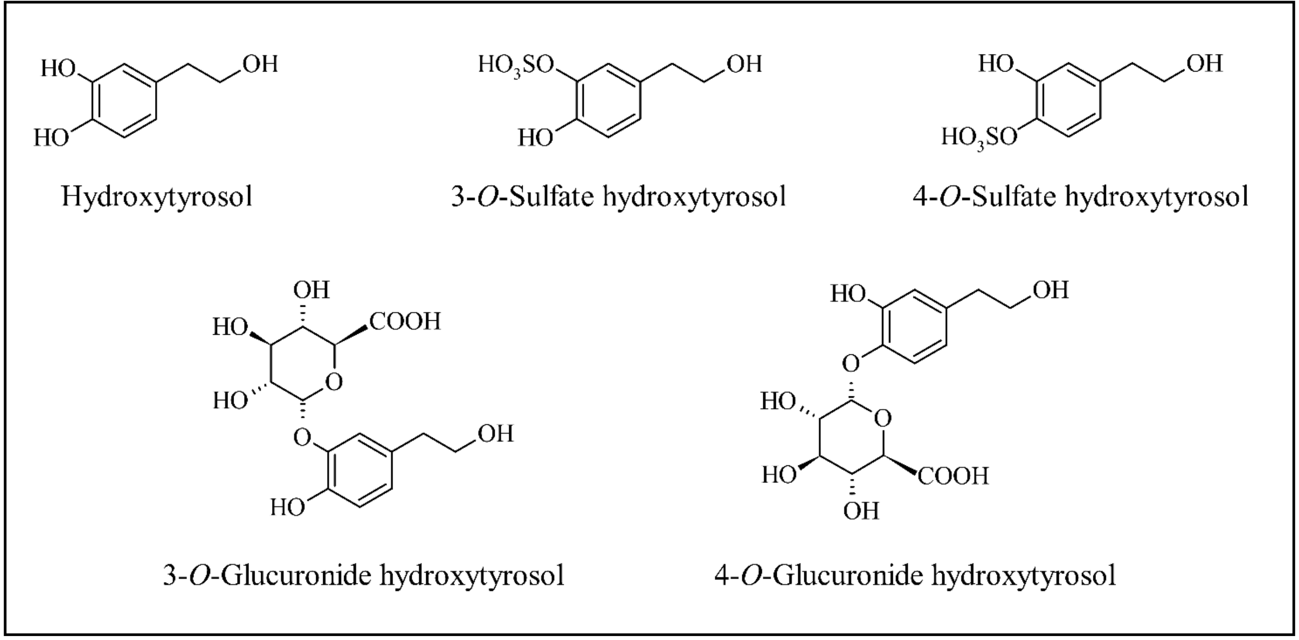

Figure S1. Chemical structures of hydroxytyrosol and its sulfate and glucuronide conjugates.
